# Phytochemical and Antiproliferative Activity of Proso Millet

**DOI:** 10.1371/journal.pone.0104058

**Published:** 2014-08-06

**Authors:** Lizhen Zhang, Ruihai Liu, Wei Niu

**Affiliations:** 1 College of Life Science, Shanxi University, Taiyuan, China; 2 Department of Food Science, Cornell University, Ithaca, New York, United States of America; 3 Shanxi Academy of Agricultural Sciences, Taiyuan, China; University of Sassari, Italy

## Abstract

The phytochemical content, antioxidant activity and antiproliferative properties of three diverse varieties of proso millet are reported. The free phenolic content ranged from 27.48 (Gumi 20) to 151.14 (Mi2504-6) mg gallic acid equiv/100 g DW. The bound phenolic content ranged from 55.95 (Gumi20) to 305.81 (Mi2504-6) mg gallic acid equiv/100 g DW. The percentage contribution of bound phenolic to the total phenolic content of genotype samples analyzed ranged between 62.08% and 67.05%. Ferulic acid and chlorogenic acid are the predominant phenolic acid found in bound fraction. Caffeic acid and *p*-coumaric acid were also detected. Syringic acid was detected only in the free fraction. The antioxidant activity was assessed using the hydrophilic peroxyl radical scavenging capacity (PSC) assay. The PSC antioxidant activity of the free fraction ranged from 57.68 (Mi2504-6) to 147.32 (Gumi20) µmol of vitamin C equiv/100 g DW. The PSC antioxidant activity of the bound fraction ranged from 95.38 (Mizao 52) to 136.48 (Gumi 20) µmol of vitamin C equiv/100 g DW. The cellular antioxidant activity (CAA) of the extract was assessed using the HepG2 model. CAA value ranged from 2.51 to 6.10 µmol equiv quercetin/100 g DW. Antiproliferative activities were also studied in vitro against MDA human breast cancer and HepG2 human liver cancer cells. Results exhibited a differential and possible selective antiproliferative property of the proso millet. These results may be used to direct the consumption of proso millet with improved health properties.

## Introduction

Proso millet (*Panicum miliaceum L.*) is an important cereal and a valuable component of the human diet, particularly in developing countries. The crop is salt-, alkali-, cold-, and drought-tolerant and can be cultivated in various types of soil and under poor growing conditions [1]. Its grains are mainly used for food in the decorticated form. Traditionally proso millet quality has been evaluated on the basis of nutritional value, such as starch [2] and crude protein contents [3]. Epidemiological studies show that increased consumption of proso millet and its products are associated with reduced risk of chronic diseases, such as elevated serum cholesterol [4], cardiovascular disease [5], type II diabetes [6], and liver injury [7]. These health benefits have been attributed in part to its unique photochemical profile. However, chemistry and biological activities, including antioxidative and antiproliferative effects of proso millet grains have not received as much attention as phytochemicals in fruits and vegetables. Therefore, the phytochemicals contents of edible proso millet need closer examination due to their potential health benefit in the prevention of chronic diseases.

Chandrasekara and Shahidi [8] reported the phenolics in millet whole grain samples, including one proso millet sample. However, millets belong to a range of different species of family *Gramineae*. Proso millet belongs to *Panicum* genus, which possesses a different phytochemical profile to those other genera in *Gramineae*. Further, proso millet germplasm collections have broad genetic variability and vary in kernel color, size, shape, and other characteristics. In China, over 8, 500 accessions (varieties and landraces) of proso millets are conserved in the National Centre for Crop Germplasm Conservation. Some varieties of proso millet seeds can be harvested from 10 to 20 weeks after planting [9], and have many different colors, such as black, red and white, and so on. As a result, a more complete analysis of the phytochemical contents and antioxidant activity of a range of diverse genetype proso millet samples are needed. Here we choosed three varieties based on their different phenotype characters, mean value of nutrients content and their widely usage in production. Therefore, the objectives of this study were to (1) determine the phytochemical profiles of total phenolics, phenloic acid composition, including both free and bound forms; (2) determine the antioxidant activity and antiproliferation in proso millet milled edible fractions; (3) determine the carotenoid content (xanthophyll, zeaxanthin, β-cryptoxanthin) of three diverse proso millet varieties.

## Materials and Methods

### Chemicals and Reagents

Methanol (MeOH), hydrochloric acid (HCl), sodium carbonate, sodium sulphate, acetone, phosphate buffered saline (PBS) were purchased from Mallinckrodt Chemicals (Phillipsburg, NJ). Folin-Ciocalteu reagent, quercetin, ascorbic acid, ferulic acid, chlorogenic acid, caffeic acid, *p*-coumaric acid and syringic acid, xanthophyll, zeaxanthin, *β*-cryptoxanthin, dichlorofluorescein- diacetate (DCFH-DA), were purchased from Sigma (St. Louis, MO). 2, 2-Azobis-amidinopropane (ABAP) was purchased from Wako Chemicals (Richmond, VA). Gallic acid was purchased from ICN Biomedical Inc. (Costa Mesa, CA). Ethyl acetate, triflouroacetic acid, and ethanol were purchased from Mallinckrodt (Paris, KS). Sodium hydroxide, hexane, acetonitrile, magnesium carbonate, tetrahydrofuran were obtained from Fisher Scientific (Pittsburgh, PA). MDA human breast cancer cell lines and HepG2 liver cancer cell lines are provided by the American Type Culture Collection (ATCC, Rockville, MD). Williams’ medium E (WME), α-MEM, Hanks’ Blanced Salt Solution (HBSS) were purchased from Gibco Life Technologies, and Fetal bovine serum (FBS) was purchased from Atlanta Biologicals (Lawrenceville, GA).

### Grain Samples and Sample Preparation

Proso millet varieties (Table 1) used in this study were provided by Shanxi Agriculture Academy. Seeds of Gumi 20, Mizao 52, Mi2504-6 were harvested from plots grown near Taiyuan, Shanxi in 2011. Gumi 20 has dark brown pigmented testa, Mizao 52 has red pigmented testa, Mi2504-6 has white pigmented testa. The three proso millet samples were dehusked to remove inedible husk and aspirated to remove husk, then milled into fine powder, screened though a 60 mesh screen and thoroughly mixed. Each sample was stored at −40°C and used within 2 weeks of milling.

**Table 1:** Description of proso millet samples.

Cultivalname	% of bran to prosomillet (g/100g)	Amylose(g/100g)	Amylopectin(g/100g)	crude protein(g/100g)	Fat(g/100g)	moisturecontent (%)
Gumi20	21.24±0.28^a^	22.03±0.74^a^	33.83±1.07^a^	10.54±0.54^b^	4.09±0.14^a^	7.96±0.14^c^
Mizao52	20.64±0.20^a^	18.85±0.53^b^	27.75±0.15^c^	11.41±0.24^a^	4.40±0.23^a^	8.35±0.18^b^
Mi2504-6	11.13±0.55^b^	20.11±0.88^b^	29.93±1.19^b^	11.62±0.19^a^	3.64±0.16^b^	9.63±0.13^a^

### Extraction of Soluble Free Phytochemical Compounds

Soluble free phenolics of proso millet samples were extracted using the method reported previously [10], [11]. Briefly, 2 g of proso millet flour was blended for 5 min in 30 mL of 80% chilled acetone (1∶8, w/w) using a Waring blender. The mixture was then centrifuged at 2, 500 g for 10 min. The supernatant was removed and the remaining pellet was again extracted with 30 mL of 80% chilled acetone two times. The supernatants were pooled and evaporated at 45°C to dryness. The final extract was diluted to 10 mL MilliQ water, filtered through a 0.45 µm filter, aliquoted into 1 mL per tube, and stored at −40°C until analysis.

### Extraction of Bound Phytochemical Compounds

Bound phytochemicals of proso millet samples were extracted using a modification of the method previously described by Adom and Liu [10]. Briefly, bound phenolics were extracted from the residue from the free extraction. The residue was first digested with 20 mL 2 M sodium hydroxide at room temperature for 1 h with shaking under nitrogen. The mixture was then neutralized with appropriate amount of concentrated hydrochloric acid. Hexanes were used to remove the lipids in the mixture. The remaining mixture was then extracted five times with ethyl acetate. The ethyl acetate fractions were pooled and evaporated at 45°C to dryness. The bound phenolics were reconstituted in 10 mL of MilliQ water, filtered through a 0.45 µm filter, aliquoted in 1 mL per tube and stored at −40°C until analysis.

### Determination of the Total Phenolic Content

The total phenolic content of each extracts was determined using the method described by Singleton et al [12] and modified by Okarter et al [13], [14]. Briefly, the appropriate dilutions of extracts were oxidized with the Folin-Ciocalteu reagent, and the reaction was neutralized with sodium carbonate. The absorbance of the resulting blue solution was measured at 760 nm in a MRX П Dynex plate reader (Dynex Technologies, Inc., Chanilly, VA) after 90 min of incubation at room temperature. Using gallic acid as a standard, the total phenolic content of samples was expressed as *mg* of gallic acid equiv/100 g of sample. Data were reported as mean ±SD for three replicates.

### Determination of Phenolic Acid Composition

The determination of the phenolic composition was conducted using an RP-HPLC method reported previously [14], [15]. Briefly, the mobile phase was delivered using a Waters 600E quaternary pump at a flow rate of 0.5 mL/min. Isocractic mobile phase was conducted with 20% acetonitrile in water adjusted to pH 2 with triflouroacetic acid. Separation of phenolic compounds was done using a Supelcosil LC-18-DB column (3 µm, 150 mm×4.6 mm). The total run time was 30 min. Twenty microlitres of sample were made in each run using a Water 717 autosampler. Phenolic compounds were detected using a Waters 2487 dural wavelength absorbance Detector. Each injection was monitored at 280 nm. Identification of each peak was confirmed using the retention time and absorbance spectrum of each pure compound. Percent recoveries were determined by spiking a known amount of pure compound into a sample and performing the same extraction and analytical procedures. The percent recovery for ferulic acid, ***p***-coumaric acid, syringic acid, caffeic acid, and cholrogenic acid were higher than 90% (n = 3). Data signals were acquired and processed using Waters Empower software (Waters Corp., Milford, MA).

### Determination of the peroxyradical scavenging capacity (PSC)

Hydrophilic peroxyradical scavenging capacity (PSC) assay was developed to determine the total antioxidant capacity of proso millet extracts based on the method described by Adom and Liu [16]. In this assay, the reaction was monitored using the fluorescent dye dichlorofluorescein. Peroxyl radicals generated by ABAP oxidize nonfluorescent dichlorofluorescein (DCFH) to fluorescent dichlorofluorescien (DCF). The degree of inhibition of DCFH oxidation by antioxidants that scavenge peroxyl radicals was used as the basis for calculating the antioxidant activity. Just prior to use in the reaction, 107 *µ*L of 2.48 mM DCFH-DA was hydrolyzed with 893 *µ*L of 1.0 mM KOH for 5 min in the dark to remove the diacetate (DA) moiety and then diluted to a total volume of 8 mL with 75 mM phosphate buffer (pH 7.4). DCFH-DA was stable to oxidation, whereas DCFH was very slowly oxidized at ambient conditions without ABAP. ABAP (200 mM) was prepared fresh in buffer, and each batch was kept at 4°C between runs and discarded after 6 h. The standard or proso millet extracts were appropriately diluted in 75 mM phosphate buffer (pH 7.4) to reach the indicated concentrations. In a run, 100 *µ*L diluted solution of the standard or proso millet extract was transferred into reaction cells in a 96-well plate, and then 100 *µ*L of DCFH was added. The 96-well plate was loaded into the plate holder for the Fluoroskan Ascent fluorescence spectrophotometer (Thermo Labsystems, Franklin, MA), and the solution in each cell was mixed by shaking at 1200 rpm for 20 s. The reaction was then initiated by adding 50 *µ*L of ABAP from the autodispenser of the equipment. The autodispenser was emptied and rinsed with fresh ABAP before each run. Each set of dilutions for a sample and control was analyzed three times in adjacent columns. The reaction was carried out at 37°C, and fluorescence generation was monitored at 485 nm excitation and 538 nm emission with the fluorescence spectrophotometer. The phosphate buffer was used for control reaction. Data were acquired with Ascent software, version 2.6 (Thermo Labsystems, Franklin, MA) running on a PC. Fluorescence values were averaged across columns for each set of dilutions. The areas under the average fluorescence-reaction time kinetic curve (AUC) for both control and samples (up to 36 min) were integrated and used for calculating peroxylradical scavenging capacity (PSC value) according to eq. 1.
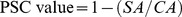
(1)Where SA is AUC for the sample or standard dilution and CA is AUC for the control reaction. Compounds or extracts inhibiting the oxidation of DCFH produced smaller SA and higher PSC values. The parameter EC_50_ was defined as the dose required to cause a 50% inhibition (PSC value = 0.5) for each pure compound or sample extract and was used as the basis for comparing different compounds or samples [16]. Results obtained for sample extract antioxidant activities were expressed as *µ*mol of vitamin C equiv/100 g of sample ±SD for triplicate analyses.

### Cell Culture

MDA human breast cancer cells were grown in α-MEM growth medium supplemented with 10% FBS, 10 mM Hepes, 50 units/mL penicillin, 50 µg/mL streptomycin, and 100 µg/mL gentamicin. HepG2 liver cancer cells were maintained in Williams’ medium E (WME) growth medium with 5% FBS, 10 mM Hepes, 50 units/mL penicillin, 50 µg/mL streptomycin, 100 µg/mL gentamicin, 5 µg/mL insulin and 0.05 µg/mL hydrocortisone. All cells were maintained at 37°C and 5% CO_2_ in an incubator as described previously [17], [18], [19]. Cells used in this study were between passages 18 and 32.

### Measurement of Cell Cytotoxicity and Inhibition of Proliferation

Cytotoxicity toward MDA and HepG2 cells were measured using the methods as described previously [18], [19]. MDA and HepG2 cells in growth media were placed in each well of a 96-well flat-bottom plate at a density of 4.0×10^4^ cells/well. After 24 h of incubation at 37°C with 5% CO_2_, the growth medium was removed, each well washed with 100 µL of PBS, and replaced by media containing different concentrations of sample tested. Control cultures received the extraction solution minus the extracts, and blank wells contained 100 µL of growth medium with no cells. After another 24 h of incubation, cytotoxicity was determined by the methylene blue assay. Cytotoxicity was determined by a 10% reduction of absorbance at 570 nm reading for each concentration compared to the control using an MRX II DYNEX spectrophotometer (DYNEX Technologies, Inc.). A minimum of three replications for each sample was used to determine the cytotoxicity.

Antiproliferative activities of proso millet extracts were measured using the methods described previously [18], [19]. MDA cells and HepG2 cells were plated in a 96 well flat-bottom plate at a concentrations of 2.5×10^4^ cells/well. After 6 h of MDA cell incubation and 4 h of HepG2 cell incubation, the growth medium was removed and media containing increasing concentrations of proso millet extracts were added to the cells. Control cultures received the extraction solution minus the proso millet extract, and blank wells contained 100 *µ*L of growth medium with no cells. After 72 h of incubation, cell proliferation was determined by the methylene blue assay. Cell proliferation was determined from the absorbance at 570 nm reading for each concentration compared to the control using an MRX II DYNEX spectrophotometer (DYNEX Technologies, Inc.).

### Cellular Antioxidant Activity

#### Extraction of Carotenoids for CAA Samples

Carotenoids were extracted using the method described by Hentschel et al [20] and modified as described previously [21]. The extraction was performed under dim lighting and all sample tubes were wrapped in lightproof paper to protect carotenoids from light-induced degradation. Briefly, 0.6 g samples was mixed with 0.06 g magnesium carbonate and extracted with 3 mL methanol/tetrahydrofuran (1∶1, v/v) solution at 75°C for 5 min in water bath, vortexed again and immediately centrifuged at 2000 *g* for 5 min. The extraction was repeated three times for complete extraction of carotenoids and the organic solvent phase was collected. The residual was rinsed twice with 2 mL hexane. The hexane and methanol/tetrahydrofuran phases were pooled and vortexed with 1.5 g sodium sulphate. The extracted solvent was evaporated to dryness under a gentle stream of nitrogen. The dry residue was re-dissolved with 1.0 mL methanol/tetrahydrofuran (1∶1, v/v), filtered through a 0.45 µm filter, stored under nitrogen at −20°C until CAA analysis within two days.

#### Quantification of CAA

The CAA of proso millet carotenoids extracts were determined using the protocol described previously [22]. Briefly, HepG2 cells were seeded at a density of 6×10^4^/well on a 96-well microplate in 100 µL of complete medium/well. Twenty-four hours after seeding, the growth medium was removed, and the wells were washed with 100 µL of PBS. Wells were then treated with 100 µL of treatment medium containing solvent control, control extracts, or tested proso millet extracts plus 25 µM DCFH-DA for 1 h. Wells were washed with 100 µL of PBS. Then 600 µM ABAP was applied to the cells in 100 µL of oxidant treatment medium (HBSS with 10 mM Hepes), and the 96-well microplate was placed into a Fluoroskan Ascent FL plate reader at 37°C. Emission at 538 nm was measured after excitation at 485 nm every 5 min for 1 h.

After blank subtraction and subtraction of the initial fluorescence values, the area under the curve for fluorescence versus time was integrated to calculate the CAA value at each concentration of proso millet as described by Wolf and Liu [22].
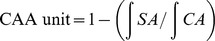
Where ∫SA is the integrated area under the sample fluorescence versus time curve and ∫CA is the integrated area from the control curve. The median effective dose (EC_50_) was determined for the proso millet extracts from the median effect plot of log (ƒa/ƒu) versus log (dose), where ƒa is the fraction affected (CAA unit) and ƒu is the fraction unaffected (1-CAA unit) by the treatment. The EC_50_ values were stated as mean± SD for triplicate sets of data obtained from the same experiment. EC_50_ values were converted to CAA values, which are expressed as micromoles of quercetin equiv/100 g sample, using the mean EC_50_ value for quercetin from three replications.

#### Determination of Carotenoid Content

Carotenoid content was determined using the method described by Hentschel et al [20] and was modified by Liu et al [21]. Briefly, the carotenoid content of each sample was determined using an RP-HPLC procedure employing a 250×4.6 mm YMC C30 column, 3 µm particle size (YMC, Waters Inc., Wilmington, NC). The mobile phase used were methanol/water (95∶5, v/v, A) and methyl tert-butyl ether (B). Isocratic elution was performed with 75% solvent A and 25% solvent B, delivered at 1.0 mL/min using a Water 515 HPLC pump (Water Corp., Milford, MA). A Waters 2487 dual wavelength absorbance detector (Waters Corps, Milford, MA) was used for UV detection of analytes at 450 nm. Data signals were acquired and processed on a PC running the Waters Millennium software, version 3.2 (1999) (Waters Corp, Milford, MA). Percent recoveries for all carotenoids were greater than 90%. The carotenoid content of each sample extract was extrapolated from a pure carotenoid standard curve. All samples were injected via a 20 µL loop and peak heights were used for all calculations. Data were expressed as mg/100 g DW.

#### Statistical Analysis

Data were expressed as the mean ± standard deviation (SD) of three measurements. One-way analysis of variance (ANOVA) was computed to determine significant differences between the means by SigmaPlot (version 11.0) software. A significant difference was defined at *p*<0.05.

## Results and Discussion

### Total Phenolic Content

**Figure 1:**
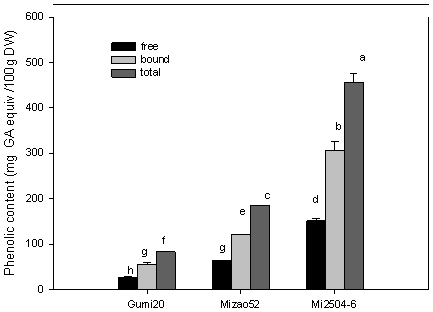
Phenolic contents of proso millet. TPCs were quantified using the Folin-Ciocalteu reagent with gallic acid as the standard. Absorbance was read at 760 nm after 90 min of reaction. Results are expressed as mg gallic acid equivalnts nm after 90 min of reaction. Results are expressed as mg gallic acid equivalnts min of reaction. Results are expressed as mg gallic acid equivalnts/100 g DW. Analyses were conducted in triplicate, with mean values shown and standard deviation depicted by the vertical bars. Column marked by the same letter are not significantly different from each other g DW. Analyses were conducted in triplicate, with mean values shown and standard deviation depicted by the vertical bars. Column marked by the same letter are not significantly different from each other (*p*<0.05).

The free and bound phenolic contents of proso millet and the percentage contribution of each fraction to the total phenolic content of different genotype samples are presented in Fig. 1, expressed as milligrams of gallic acid equiv/100 g DW. The free phenolic content ranged from 27.48 (Gumi 20) to 151.14 (Mi2504-6) mg gallic acid equiv/100 g DW. The percentage contribution of free phenolic to the total phenolic was between 32.93 and 34.72%. The bound phenolic content ranged from 55.95 (Gumi20) to 305.81 (Mi2504-6) mg gallic acid equivalent per 100 g DW. The percentage contribution of bound phenolic to the total ranged between 65.28 and 67.05%. The total phenolic content ranged from 83.44 (Gumi 20) to 456.95 (Mi2504-6) mg gallic acid equivalent per 100 g DW. Those results indicated that bound phenolic content of proso millet was significantly higher than that of free phenolic content.

Similar to other crops [14] and fruits [15], an influence of genetic on the content of phenolics was observed in this study. The free phenolic content, bound phenolic content and total phenolic content were significantly different between three proso millet varieties (*p*<0.05). Mi2504-6 had the highest phenolic content among the three proso millet varieties, followed by Mizao52. Gumi20 had the lowest phenolic content among the three proso millet varieties. In this study, there was a 5.5-fold difference in free phenolic content, bound phenolic content and total phenolic content between the highest and lowest ranked varieties. Chandrasekara and Shahidi [23] presented the total phenolic of millets with dark brown pigmented testa and pericarp is higher than those with white or yellow testa and pericarp. However, our results showed the phenolic content of different proso millet depends mainly on the varietal differences, not on millet type and color.

### Phenolic acid composition

Results for free and bound phenolic acid composition of the tested proso millet varieties are presented in Table 2. Chlorogenic acid was the predominant phenolic acid found in each variety of proso millet tested and was found both in the free and bound forms. No free chlorogenic acid was detected in Mi2604-6. Free chlorogenic acid content ranged from 5.99 (Mizao52) to 6.38 (Gumi20) mg chlorogenic acid/100 g DW. Bound chlorogenic acid contents ranged from 18.80 (Mi2504-6) to 20.69 (Gumi20) mg chlorogenic acid/100 g DW. Total chlorogenic acid content ranged from 18.80 (Mi2504-6) to 27.06 (Gumi20) mg chlorogenic acid/100 g DW.

**Table 2:** Phenolic acid composition of three diverse varieties of proso millet.

	Free	Bound	Total
Chlorogenic acid			
**Gumi 20**	6.38±0.38^a^ (23.54)	20.69±0.24^a^ (76.46)	27.06±0.28^a^
**Mizao 52**	5.99±0.03^a^ (23.42)	20.59±5.96^a^ (76.58)	26.57±5.96^a^
**Mi2504-6**	nd	18.80±1.80^a^ (100)	18.80±1.80^a^
Syringic acid			
**Gumi 20**	3.05±0.23^a^	nd	3.05±0.23^a^
**Mizao 52**	0.74±0.21^b^	nd	0.74±0.21^b^
**Mi2504-6**	0.48±0.19^b^	nd	0.48±0.19^b^
Caffeic acid			
**Gumi 20**	3.64±0.02^a^ (48.17)	3.91±0.01^a^ (51.83)	7.55±0.02^a^
**Mizao 52**	nd	3.98±0.26^a^ (100)	3.98±0.26^b^
**Mi2504-6**	nd	4.36±0.42^a^ (100)	4.36±0.42^b^
ρ-coumaric acid			
**Gumi 20**	3.94±0.15^a^ (47.16)	4.41±0.64^b^ (52.84)	8.35±0.75^a^
**Mizao 52**	nd	5.18±0.68^ab^ (100)	5.18±0.68^b^
**Mi2504-6**	nd	6.08±0.08^a^ (100)	6.08±0.08^b^
Ferulic acid			
**Gumi 20**	nd	14.68±1.30^b^ (100)	14.68±1.30^b^
**Mizao 52**	nd	23.56±0.24^a^ (100)	23.56±0.24^a^
**Mi2504-6**	nd	24.18±0.10^a^ (100)	24.18±0.10^a^

Values expressed as mg phenolic acid/100 g DW (mean±SD, n = 3). Percent contribution to total phenolic acid content is in parentheses. Values with no letters in common within each column are significantly different (p<0.05); nd-not detected.

Syring acid was found only existed in the free form in the tested proso millet varieties. Free syring acid contents ranged from 0.48 (Mi2504-6) to 3.05 (Gumi20) mg syringic acid/100 g DW.

Caffeic acid was found existed in the bound form in all tested varieties. Free caffeic acid was only found in Gumi20 and the content was 3.64 mg caffeic acid/100 g DW. Bound caffeic acid contents ranged from 3.91 (Gumi20) to 4.36 (Mi2504-6) mg caffeic acid/100 g DW.

ρ-Coumaric acid was found existed in the bound form in all tested varieties. Free ρ-coumaric acid was only found in Gumi20 and the content was 3.94 mg ρ-coumaric acid/100 g DW. Bound ρ-coumaric acid contents ranged from 4.41 (Gumi20) to 6.08 (Mi2504-6) mg ρ-coumaric acid/100 g DW.

Ferulic acid is another the predominant phenolic acid found in all tested edible proso millet varieties and was found existed only in the bound form. The bound ferulic acid contents ranged from 14.68 (Gumi20) to 24.18 (Mi2504-6) mg ferulic acid/100 g DW.

Phenolic acids are hydroxylated compounds that are derived from benzoic acid and cinnamic acid. We found the hydroxycinnamic acid derivatives are more prevalent than the hydroxybenzoic acid derivatives in proso millet. The hydroxycinnamic acids found in edible proso millet include chlorogenic acid, ferulic acid, caffeic acid and ρ-coumaric acid. The dexydroxybenzoic acid found in edible proso millet was syring acid, and only found existed in the free form.

RP-HPLC analysis revealed that the content of each hydroxycinnamic acid in the bound fraction was higher than that in the free fraction of three varieties examined in this study. Chandrasekara and Shahidi [9] also reported the bound ferulic acid and ρ-coumaric content of proso millet higher than its soluble counterparts.

Plant-derived phenolic acids received considerable interest because of their potential antioxidant and anticancer properties. McDonough and Rooney [24] reported the ferulic acid, coumaric acid, cinnamic and gentisic acid contents of finger millet, pearl millet, teff millet, fonio millet and foxtail millet. Chandrasekara and Shahidi [23] reported the ferulic acid and ρ-coumaric acid contents of proso millet. Ferulic acid and chlorogenic acid are the predominant phenolic acid found in the bound form. Here, chlorogenic acid, caffeic acid and syringic acid are found in the proso millet for the first time.

### Carotenoid content

Results for carotenoid content of the tested proso millet varieties are presented in Table 3. Xanthophyll content ranged from 0.49 (Mizao52) to 1.51 (Mi2504-6) mg/100 g DW. Zeaxanthin ranged from 1.60 (Gumi20) to 1.68 (Mi2504-6) mg/100 g DW. β-cryptoxanthin was not detected in three variety tested. In this study, xanthophyll and zeaxanthin were significantly different among the three variety tested. Reports on carotenoid in proso millet variety are scanty. Asharani et al [25] reported the total carotenoid content of five different cultivars and the total carotenoid content ranged from 0.518 to 0.249 mg/100 g. However the xanthophyll, zeaxanthin, β-cryptoxanthin content of each sample was not reported. Compared with other grains, carotenoids are rather abound in proso millet. Kean et al [26] reported the carotenoids in sorghum grains. The lutein (xanthophyll) concentrations ranged from 0.149 to 0.301 mg/kg wet weight. The zeaxanthin concentration ranged from 0.126 to 0.362 mg/kg wet weight.

**Table 3:** Carotenoid (xanthophyll, zeaxanthin, β-cryptoxanthin) content and distribution of proso millet varieties (mean±SD, n = 3).

	xanthophyll	zeaxanthin	β-cryptoxanthin
Gumi20	0.50±13.8^b^	1.60±0.4^b^	nd
Mizao52	0.49±20.4^b^	1.61±2.1^b^	nd
M2504-6	1.51±340.3^a^	1.68±16.9^a^	nd

Values expressed as mg/100 g DW. Values with no letters in common within each column are significantly different (p<0.05); nd-not detected.

Hulshof et al [27] reported the carotenoids in corn. The lutein (xanthophyll) concentrations ranged from <0.1 mg/100 g to 2.047 mg/100 g. The zeaxanthin concentration ranged from 0.129 to 2.070 mg/100 g. Kimura et al [28] reported the carotenoids in maize. The lutein (xanthophyll) concentrations ranged from 0.148 to 0.360 mg/100 g. The zeaxanthin concentration ranged from 0.401 to 0.565 mg/100 g. Our results indicate that high carotenoid proso millet varieties suitable for production of functional foods for populations at risk of vitamin.

### PSC Antioxidant Activity

**Figure 2:**
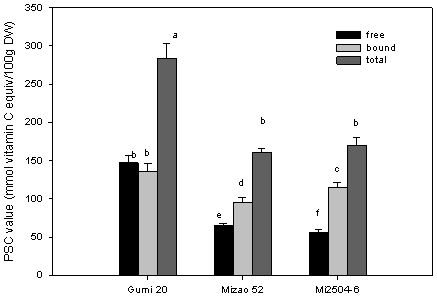
PSC antioxidant activity of proso millet. Peroxyl radical scavenging capacity assay is based on the degree of inhabitation of dichlorofluorescin oxidation by antioxidants that scavenge peroxyl radicals, generated from thermal degradation of 2, 2′azobis (amidinopropane). The median effective concentration (EC_50_) was defined as the dose required to cause a 50% inhibition for each sample extract. Results obtained for sample extract antioxidant activities were expressed as micromoles of vitamin C equivalents/100 g DW. Analyses were conducted in triplicate, with mean values shown and standard deviation depicted by the vertical bars. Column marked by the same letter are not significantly different from each other g DW. Analyses were conducted in triplicate, with mean values shown and standard deviation depicted by the vertical bars. Column marked by the same letter are not significantly different from each other (*p*<0.05).

Results for PSC antioxidant activity of tested proso milllet varieties are presented in Figure 2. The free PSC antioxidant activity ranged from 57.68 (Mi2504-6) to 147.32 (Gumi20) µmol of vitamin C equiv/g. The bound PSC antioxidant activity ranged from 95.38 (Mizao 52) to 136.48 (Gumi 20) µmol of vitamin C equiv/g. The total PSC antioxidant activity of the wheat samples ranged from 161.26 (Mizao52) to 283.82 (Gumi 20) µmol of vitamin C equiv/g.

Asharni et al [25] quantified the total antioxidant activities of the edible flours of proso millet using the phosphomolybdenum reagent and found the antioxidant activity of proso millet varieties ranged from 4.5 to 5.7 mM tocopherol equivalent/g. Chandrasekara and Shahidi [23] evaluated the antioxidant activities of the proso millet on the basis of scavenging capacity of 2,2-diphenyl-1-picrylhydrazyl (DPPH^−^) radicals and reactive oxygen species (ROS) in vitro chemical assays.

### Cellular Antioxidant Activity

**Figure 3:**
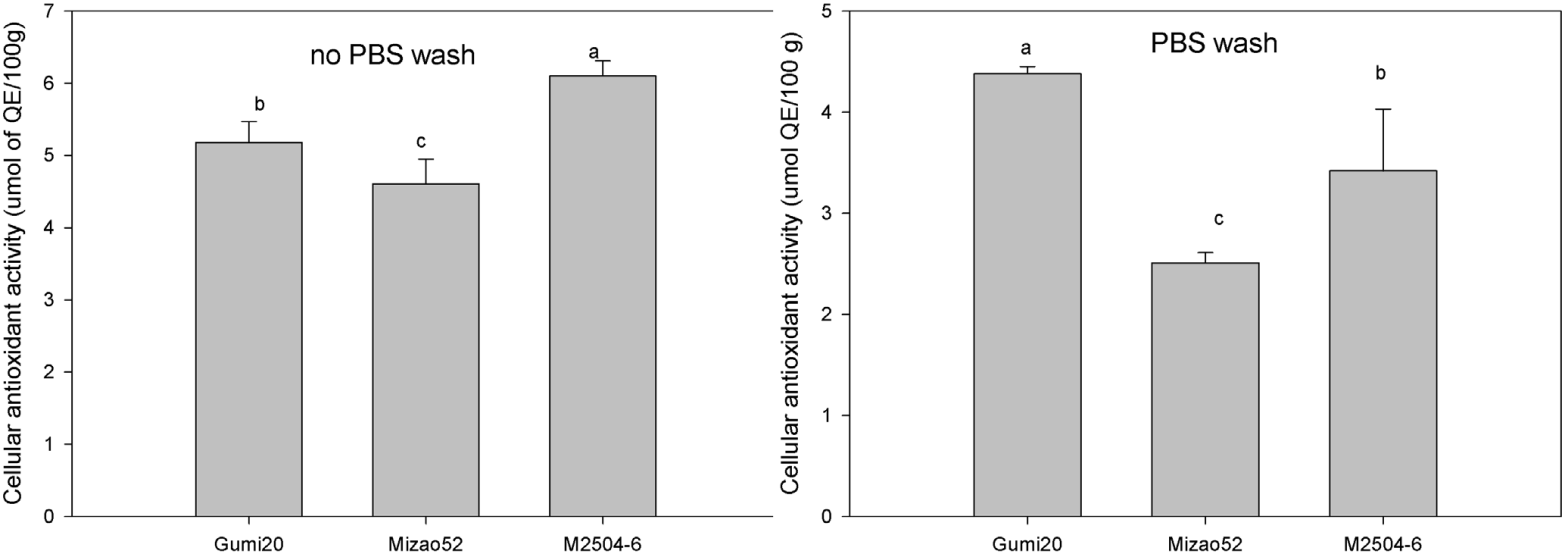
Cellular antioxidant activity of proso millet. Cellular antioxidant activity (CAA) assay is based on the ability of compounds to prevent the formation of DCF by 2,2′-azobis (2-amidinopropane) dihydrochloride (ABAP)-generated peroxyl radicals in human hepatocarcinoma HepG2 cells. Values are based on triplicate tests, with mean values shown and standard deviation depicted by the vertical bars. Column marked by the same letter are not significantly different from each other (p<0.05).

The cellular antioxidant activities of the carotenoids extracts of proso millet were measured using the CAA assay. Both protocols with and without PBS wash were used to measure the cellular antioxidant activity. The EC_50_ for the extracts are presented in Table 4. The EC_50_ values were converted to CAA values, expressed as micromoles of quercetin equivalent per 100 g of dried proso weight (Fig. 3). When a PBS wash was done between antioxidant and ABAP treatments, the PBS will remove compounds that are loosely associated with the membrane. So for all varieties proso millet tested in our study, the antioxidant quality was lower and the EC_50_ value was higher using the protocol with PBS wash (p<0.05). Significant differences in cellular antioxidant activities of extracts were observed among the three proso millets. In the protocol without PBS wash, Mi2504-6 had the greatest cellular antioxidant activity, with a CAA value of 6.10 µmol quercetin equivalent/100 g DW, followed by Gumi20 and Mizao52, which showed CAA values of 5.18 and 4.16 µmol of quercetin equivalent/100 g DW, respectively. In the protocol with PBS wash, Gumi20 had the greatest cellular antioxidant activity, with a CAA value of 4.38 µmol of quercetin equivalent/100 g DW, followed byMi2504-6 and Mizao52, which had CAA values of 3.42 and 2.51 µmol of quercetin equivalent/100 g DW, respectively.

**Table 4:** Cellular antioxidant activities of proso millet expressed as EC50 and CAA values (Mean±SD, n = 3).

Proso millet	Without PBS wash		With PBS wash	
	EC_50_ (mg/mL)	CAA (µmol of QE/100g)	EC_50_ (mg/mL)	CAA (µmol of QE/100g)
Gumi20	167.57±14.47^a^	5.18±0.29^b^	197.08±2.99^a^	4.38±0.07^a^
Mizao52	187.99±14.50^a^	4.61±0.34^c^	338±14.06^b^	2.51±0.10^c^
M2504-6	142.14±5.11^b^	6.10±0.21^a^	210.80±19.40^a^	3.42±0.61^b^

Values with no letters in common within each column are significantly different (p<0.05).

The cell-based antioxidant assay may be regarded as a more biological relevant method because it accounts for some aspects of uptake, metabolism, and location of antioxidant compounds within cells [22]. The cellular antioxidant activities of varieties were first investigated here. When compared to CAA values of fruits [29], vegetables [30], legumes [31], the carotenoids extracts of proso millet exhibited similar CAA values. These results indicated that proso millet also has strong cellular antioxidant activity.

Both water-soluble and lipid-soluble components of foods are important in combating specific types of radicals and diseases. However, most of the data presented in the literature have been on antioxidant activities of water-soluble food extracts. Here the cellular antioxidant activity of carotenoids extract of proso millet is reported. Proso millets being the primary food in Asian and African countries will provide with a good proportion of carotenoids in the diet. Hence, detailed investigations on carotenoids of proso millets will be very useful for their utilization in health foods.

### Antiproliferation Activity

**Figure 4:**
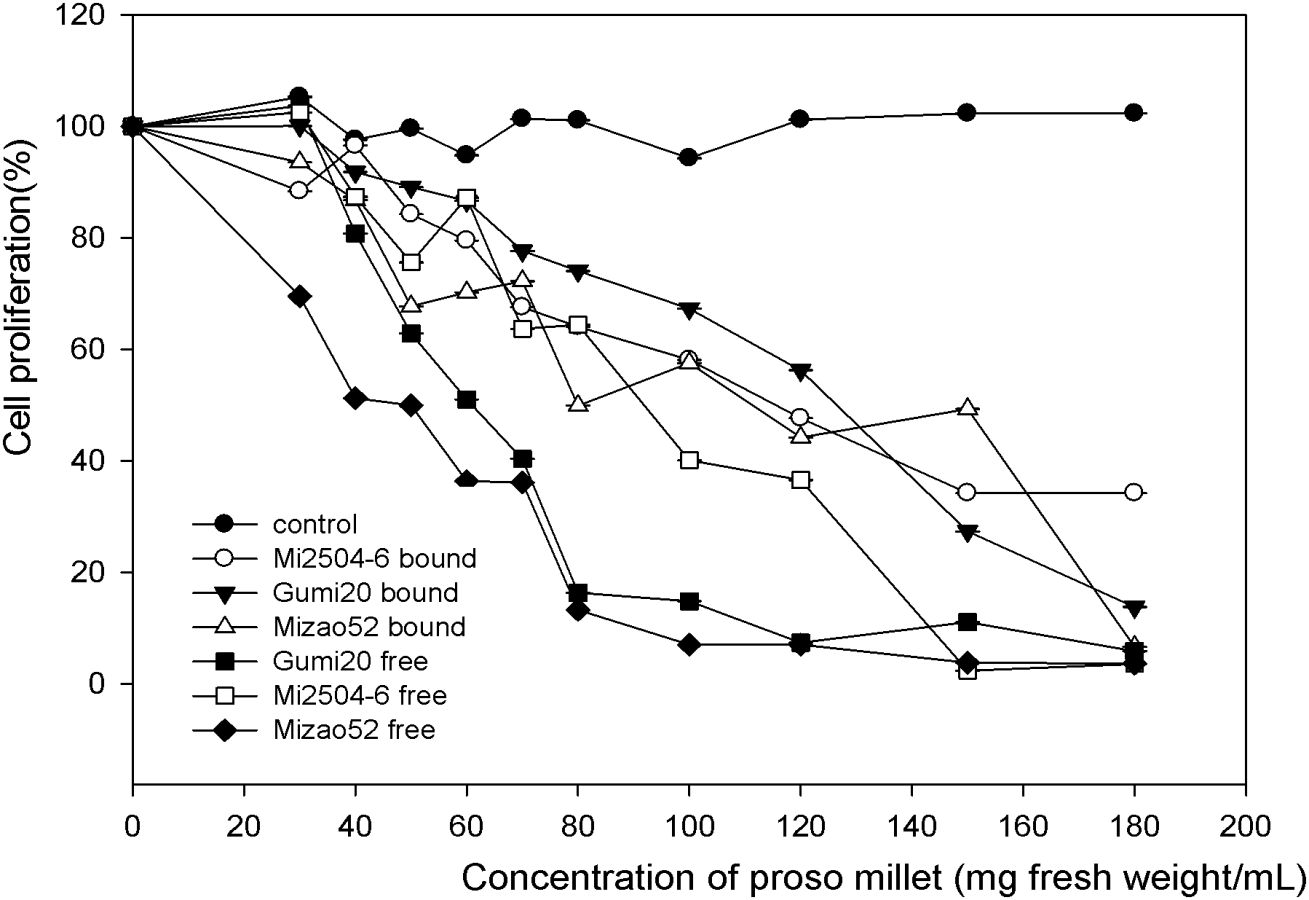
Percentage inhibition of MDA proliferation by proso millet extract. MDA cell (2.5×10^4^/mL) were incubated for 6 h to allow sufficient attachment. For the lower level treatment, the initial concentration for samples was 30 mg DW h to allow sufficient attachment. For the lower level treatment, the initial concentration for samples was 30 mg DW mg DW/mL, whereas the high concentration was 180 mg DW mg DW/mL. After 72 h of incubation, cell proliferation was determined by the methylene blue assay from the absorbance at 570 nm for each concentration compared to the control. Data were reported as mean h of incubation, cell proliferation was determined by the methylene blue assay from the absorbance at 570 nm for each concentration compared to the control. Data were reported as mean nm for each concentration compared to the control. Data were reported as mean ± SD for three replications.

**Figure 5:**
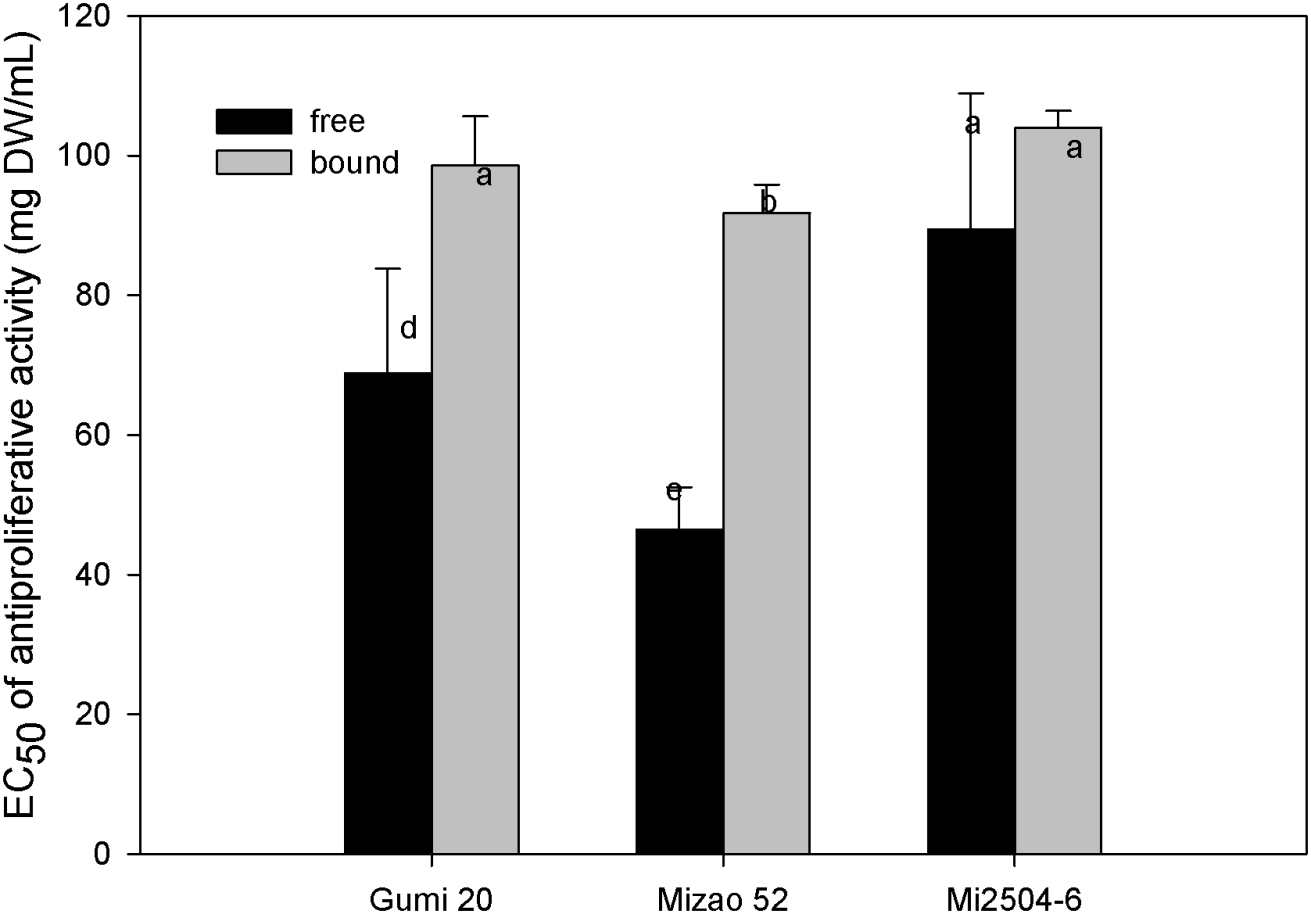
Antiproliferative activities of proso millet against MAD human breast cancer. The antiproliferative activities of proso millets against MAD cells are expressed as the median effective dose (EC_50_). Values are based on triplicate tests, with mean values shown and standard deviation depicted by the vertical bars. Column marked by the same letter are not significantly different from each other (*p*<0.05).

The inhibiting effect of proso millet extracts toward the growth of MDA human breast cells in vitro is presented in Figure 4 and Fig. 5, respectively. The antiproliferative activities of proso millets are expressed as the median effective dose (EC_50_), with a lower EC_50_ value signifying a higher antiproliferative acitivity. The free extracts of edible proso millet showed relatively higher antiproliferative activities towards MDA cells than bound extracts in a dose-dependent manner. The free extract of Mizao 52 had the highest antiproliferative activity with the lowest EC_50_ of 46.47 mg/mL, followed by the free extract of Gumi20 (68.88 mg/mL), the free extract of Mi2504-6 (89.45 mg/mL). The bound extract of Mizao 52 had the antiproliferative activity with the EC_50_ of 91.78 mg/mL, followed by Gumi 20 (98.65 mg/mL), Mi2504-6 (104.01 mg/mL).

**Figure 6:**
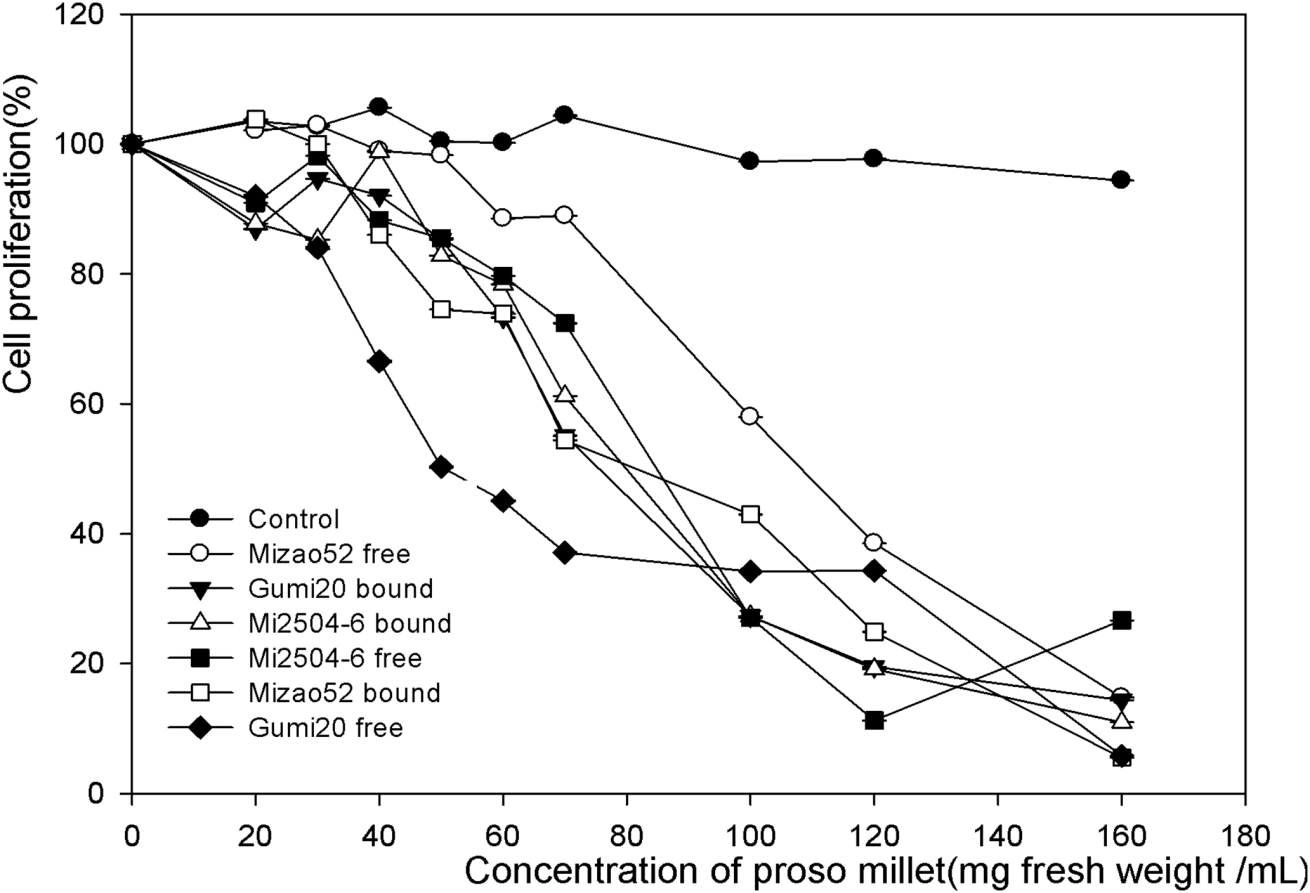
Percentage inhibition of HepG2 proliferation by proso millet extract. HepG2 cells (2.5×10^4^/mL) were incubated for 4 h to allow sufficient attachment. For the lower level treatment, the initial concentration for samples was 20 mg DW h to allow sufficient attachment. For the lower level treatment, the initial concentration for samples was 20 mg DW mg DW/mL, whereas the high concentration was 160 mg DW mg DW/mL. After 72 h of incubation, cell proliferation was determined by the methylene blue assay from the absorbance at 570 nm for each concentration compared to the control. Data were reported as mean h of incubation, cell proliferation was determined by the methylene blue assay from the absorbance at 570 nm for each concentration compared to the control. Data were reported as mean nm for each concentration compared to the control. Data were reported as mean ± SD for three replications.

**Figure 7:**
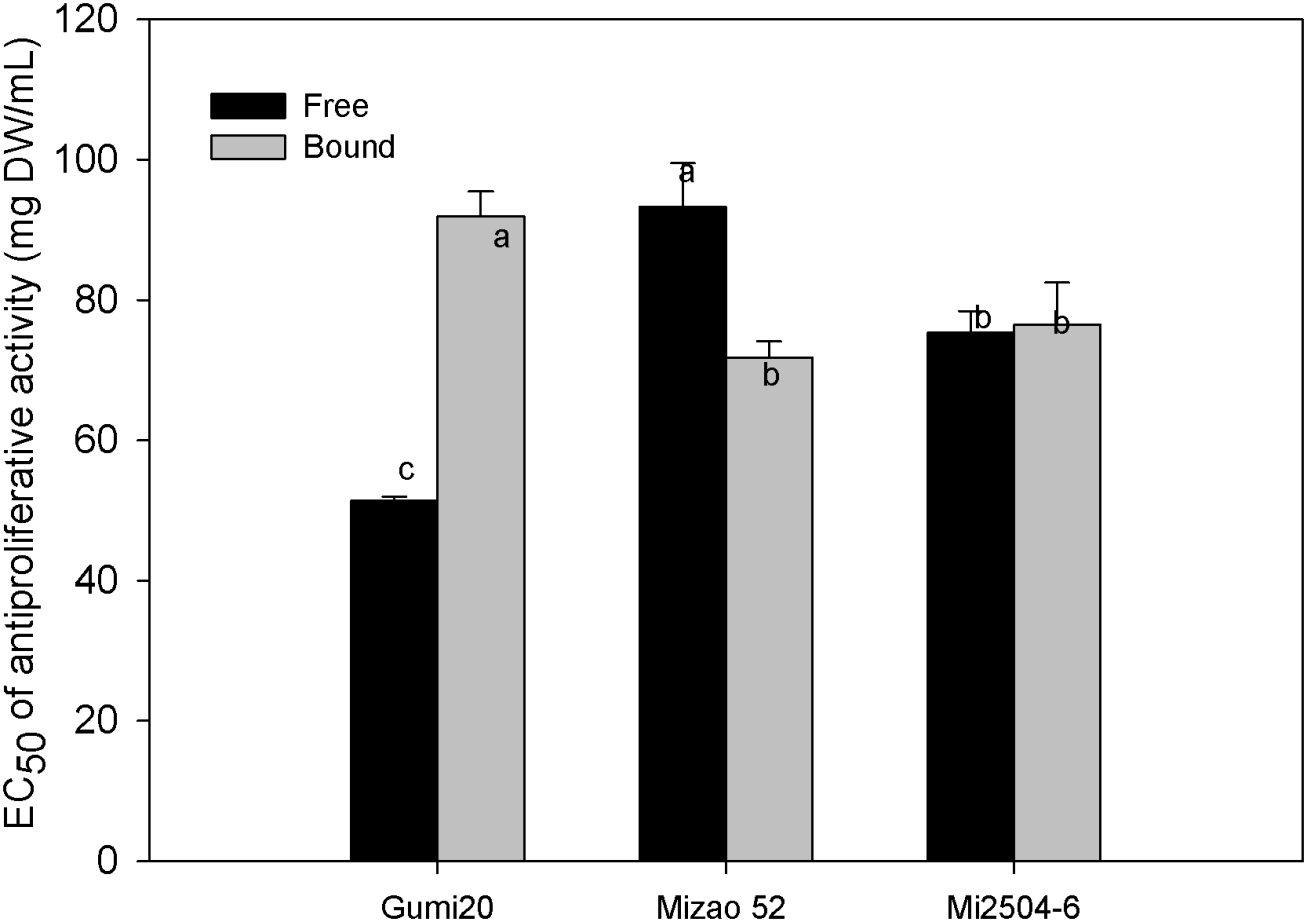
Antiproliferative activities of proso millet against HepG2 human liver cancer cells. The antiproliferative activity of proso millets against HepG2 cells is expressed as the median effective dose (EC_50_), with a lower EC_50_ value signifying a higher antiproliferative acitivity. Values are based on triplicate tests, with mean values shown and standard deviation depicted by the vertical bars. Column marked by the same letter are not significantly different from each other (*p*<0.05).

Antiproliferative activity of proso millet extracts on the growth of human HepG2 liver cancer cells in vitro is summarized in Figure 6 and Figure 7. Mizao52, Gumi20 and Mi2504-6 showed relatively potent antiproliferative activities on HepG2 cell growth in a dose-dependent manner. The free extract of Gumi20 had the highest antiproliferative activity with the lowest EC_50_ of 51.37 mg/mL, followed by bound extract of Mizao52 (71.83 mg/mL), free extract of Mi2504-6 (75.34 mg/mL), bound extract of Mi2504-6 (76.45 mg/mL). The free extract of Mizao 52 (93.28) and the bound extract of Gumi20 (91.92) had the lowest antiproliferative activity with the highestest EC_50._

In the tests, there was no significant cytotoxicity of both free and bound proso millet extracts against MDA and HepG2 cells up to 120 mg/mL. This suggested that the antiproliferative was not caused by the cytotoxicity.

Traditionally, cereals and its ingredients are accepted as functional foods and nutraceuticals because they provide antioxidants required for human health [32]. In recent years, studies have shown an association between increased consumption of whole-grain cereals and reduced risk of cancers. Several studies have highlighted the contribution of the phenolic acids to their anticancer effect. Other researchers’ work also lends support to this hypothesis. Kampa et al [33] reported the antiproliferative activity of caffeic acid, ferulic acid and syringic acid against the T47D human breast cancer cells. Birgit et al [34] reported the antiproliferative activity of ferulic acid and ρ-coumaric acid against the Caco-2 cells. However, further phytochemical and biological investigation is needed to elucidate the active compounds that are responsible for the antiproliferative activity proso millet.

In summary, the present work revealed cellular antioxidant and antiproliferative properties proso millet for the first time. The contents of phenolic acids and antioxidant activity of diverse varieties of proso millet are reported. The bound fraction contributed about 65% of the total phenolic content of the tested proso millet varieties. Proso millet is also rich in bioactive phytochemicals, including ferulic acid, chlorogenic acid, syringic acid, caffeic acid and *p*-coumaric, suggesting its potential benefits to human heath.
